# Treatment Patterns by Physiologic Age in Older Adults with Early-Stage Breast Cancer: A Single Institution Retrospective Study

**DOI:** 10.3390/jcm14217853

**Published:** 2025-11-05

**Authors:** Eliza H. Lorentzen, Yu-Jen Chen, Maria Harvey, Christina A. Minami

**Affiliations:** 1 Department of Surgery, Brigham and Women’s Hospital, Boston, MA 02115, USAmeharvey@bwh.harvard.edu (M.H.); 2 Center for Surgery and Public Health, Brigham and Women’s Hospital, Boston, MA 02120, USA; ychen148@bwh.harvard.edu

**Keywords:** breast cancer, frailty, life expectancy, geriatrics, overtreatment, undertreatment

## Abstract

**Background/Objectives**: Older adults with breast cancer may suffer from over- and undertreatment if intensity of therapy does not align with their physiologic age. We sought to evaluate the association between physiologic age, chronologic age, and treatment patterns in women ≥ 70 years with non-metastatic breast cancer. **Methods**: Patients ≥ 70 diagnosed with non-metastatic breast cancer 10/2021–3/2024 who had received surgical therapy and frailty (Geriatric-8) and life expectancy (Schonberg index) screening at our institution were identified from our institutional database. Descriptive analyses were run using chi-square tests of proportion. In the largest subgroup (patients with hormone receptor-positive (HR+)/human epidermal growth factor receptor 2-negative (HER2)-disease), multivariate logistic regression adjusting for patient- and disease-level characteristics was used to assess the relationship between life expectancy < 10 years and the omission of sentinel lymph node biopsy (SLNB) and radiation therapy (RT). **Results**: Of 272 patients, 104 (38.2%) screened positive for frailty and 64 (23.5%) had a life expectancy of <10 years. On bivariate analysis, a higher proportion of frail patients (44 (42.3%) had a life expectancy < 10 years, while 20 (11.9%) robust patients had a life expectancy < 10 years (*p* < 0.001). Most patients (226, 83.1%) had HR+/HER-2 negative disease; 10 (3.7%) had HER2+ disease; and 33 (12.1%) had triple-negative breast cancer (TNBC) (*p* < 0.001). Life expectancy was not significantly associated with omission of SLNB (life expectancy < 10 years: reference; life expectancy ≥ 10 years: OR 0.81 95% CI [0.20–3.28]) or RT (life expectancy < 10 years: reference; life expectancy ≥ 10 years: OR 1.14, 95% CI 0.44–2.93]) in patients with stage I–II HR+/HER-2− disease on adjusted analysis. **Conclusions**: While patients at risk for frailty and limited life expectancy are relatively common in our population, these measures may not significantly influence patient and clinician treatment decision making. Future efforts to tailor therapy by measures of physiologic age are needed.

## 1. Introduction

With the aging of the world’s population comes an increase in breast cancer in older adults [1,2]. Treating these older adults can be nuanced, as the heterogeneity of the aging process makes “guideline-concordant care” appropriate for some older adults but inappropriate in others. The concepts of overtreatment and undertreatment are defined by a given patient’s physiologic age and individual vulnerabilities [3], and although the American Society of Clinical Oncology and the International Society of Geriatric Oncology recommend the use of objective clinical geriatric assessments to help to guide treatment decision making [4,5], these geriatric-specific data are not widely available in national datasets. Understanding treatment patterns within the context of geriatric-specific considerations, such as frailty and limited life expectancy, remains underexplored.

Overtreatment and undertreatment may be difficult to define across a population, as an individual’s vulnerabilities need to be considered when making tailored treatment decisions. However, when considering subpopulations, examining patients by tumor subtype, there are existing data that suggest that certain practices may be considered overtreatment or undertreatment. For instance, in hormone receptor-positive (HR+), human epidermal growth factor receptor-2 (HER-2)-negative disease, randomized controlled trial data with >10 years of follow-up show no survival decrement with the omission of sentinel lymph node biopsy (SLNB) [6,7] and radiation therapy (RT) [8,9]. In this context, overtreatment may be suspected when patients with early-stage HR+/HER2- negative disease with a <10 year life expectancy still receive these therapies. There are no similar de-escalation data in patients with HER-2−positive or triple negative breast cancer (TBNC), where the standard of care across the life span remains surgery, systemic therapies including chemotherapy, and often RT as well. Undertreatment may thus be suspected in patients who have a longer life expectancy who do not receive these therapies. Defining overtreatment and undertreatment in patients with HR+/HER2- disease with life expectancies ≥ 10 years, or those with HER-2+ or TNBC with limited life expectancies, is much less clear and must be determined on an individual basis after multidisciplinary discussion. The multitude of reasonable treatment options available to older adult patients and the need to tailor treatment to physiology can result in wide variation in the treatment received by this demographic [10,11]. The objectives of this study were thus to (1) determine the prevalence of frailty and limited life expectancy and (2) examine the association between life expectancy, chronologic age, and treatment patterns in women ≥ 70 years with non-metastatic breast cancer.

## 2. Materials and Methods

Patients ≥ 70 who were diagnosed from 10/2021 to 3/2024 with non-metastatic breast cancer with geriatric-specific data were identified from the Dana–Farber/Brigham Cancer Center (DF/BCC) Clinical Operations Quality Database (COQD). The COQD is a prospectively maintained clinical database within the Division of Breast Surgery containing patient- and treatment-related data from patients undergoing surgery at our institution dating back to 2016. The database began to house geriatric-specific data for patients ≥ 70 years as of 10/2021, when the Division of Breast Surgery began a frailty and limited life expectancy screening program. Patients ≥ 70 years presenting for a treatment consultation with a member of the Division of Breast Surgery are screened by clinical staff for frailty using the Geriatric-8 [12] and for limited life expectancy using the Schonberg Index [13]. The Geriatric-8 is a validated 8-item instrument that assesses multiple domains, including mood, nutrition, polypharmacy, and self-reported health, and that allows clinicians to determine whether patients are at risk for frailty (score ≤ 14). The Schonberg index, similarly, is a validated index that calculates mortality risk based on multiple geriatric-specific domains and allows patients to be categorized as having a life expectancy of <5 years, 5–10 years, 10–14 years, or >14 years. For the purposes of this analysis, life expectancy was dichotomized as <10 years or ≥10 years.

**Figure 1:**
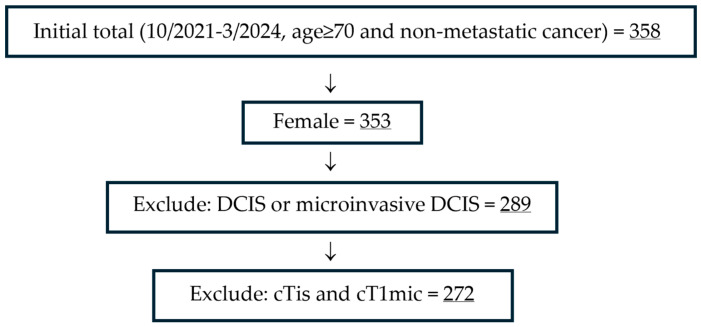
Study Flow Diagram.

Women who were 70 years and older, with non-metastatic invasive cancer, diagnosed between 10/2021 and 3/2024 were included. Patients with ductal carcinoma in situ (DCIS) or microinvasive DCIS were excluded (Figure 1).

Chi-square tests of proportions were run to determine significant differences in patient- and treatment-level factors by life expectancy and to explore the relationship between chronological age, frailty, and life expectancy. Treatment patterns were stratified by tumor subtype: HR+/HER2−, HER-2+ (including HR−/HER2+ and HR+/HER-2+ disease), and TNBC. Rates of SLNB and RT receipt in patients with Stage I–II HR+/HER2- disease were also examined. Multivariable logistic regression models adjusting for patient- and disease-level characteristics and applying Firth’s bias correction were run to assess factors associated with SLNB and RT receipt in this subgroup. Similarly, models were run for receipt of multi-modal therapy, defined as receipt of surgery and chemotherapy and/or HER-2−directed therapy in the HER2+/TNBC subgroup. Models were initially run, including chronologic age, life expectancy, and frailty. Given the potential for collinearity between these covariates, sensitivity analyses were performed without chronologic age and frailty to ensure robustness of the results. Adjusted analyses in other subgroups were not pursued, given the small populations. All analyses were performed using SAS software, v.9.4 (SAS Institute, Cary, NC, USA).

## 3. Results

Of 272 patients, only 57 (21%) were >80 years old, but 104 (38.2%) screened positive for frailty and 64 (23.5%) had a life expectancy of <10 years (Table 1). On bivariate analysis, a lower proportion of patients ≥ 80 years of age were at risk of being frail (56.1%), compared with 68.8% of those with a life expectancy < 10 years. The majority of patients (226, 83.1%) had HR+/HER-2− disease, while 10 (3.7%) had HER-2+ disease, and 33 (12.1%) had TNBC.

**Figure 2:**
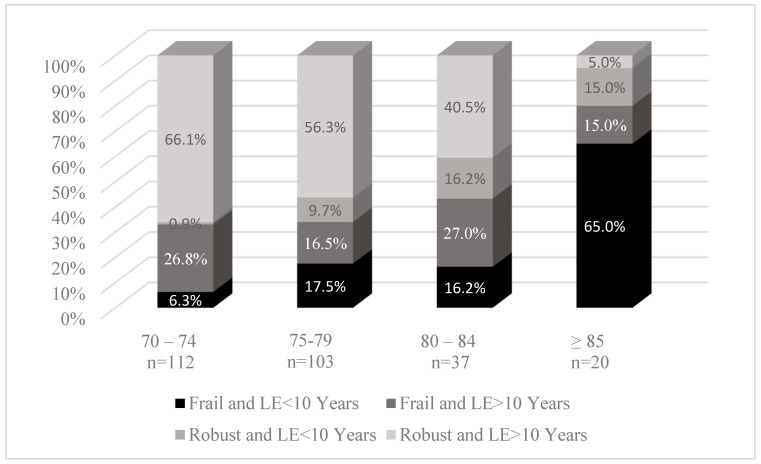
Patients 70 years and older with early-stage breast cancer by frailty and life expectancy status. Abbreviations: LE: life expectancy.

Rates of frailty and life expectancy by chronological age did differ significantly (Figure 2). Most patients 70–74 years of age were both robust and had a life expectancy ≥ 10 years (66.1%), but this proportion decreased with increasing age. In patients ≥ 85, 65% were both frail and had a life expectancy < 10 years. In the entire population, there were also small proportions of patients (15–27%) who were frail but had a life expectancy ≥ 10 years, as well as a similarly small proportion of patients who were robust and had a life expectancy < 10 years (0.9–15%).

In the largest subgroup of patients (those with HR+/HER-2− disease), the majority underwent lumpectomy alone (75.7%) (Table 2). Only 19 (8.4%) patients had axillary lymph node surgery with their lumpectomy, and only 31 (13.7%) underwent mastectomy. Of the patients who had a mastectomy, the majority (27, 87.1%) did not have reconstruction; of those with a life expectancy <10 years, none underwent reconstruction. Differences by life expectancy for these surgical treatments were not statistically significant (*p* = 0.78), nor were those by chronologic age (*p* = 0.37). Adjuvant radiation therapy (RT) was used in the minority of patients (22.1%); of these, most of these patients had a life expectancy ≥ 10 years (86%) or were <80 years of age (92%). Neoadjuvant or adjuvant chemotherapy was received by only 10 patients (4.4%), and neoadjuvant endocrine therapy was used in only 6.2%. Adjuvant CDK 4/6 inhibitors paired with endocrine therapy (ET) were used in only 5 patients (2.2%); all patients receiving these therapies had a life expectancy of ≥10 years and were <80 years of age.

In patients with HER2+ or TNBC, the rates of neoadjuvant chemotherapy were much higher, with 44.3% of patients receiving neoadjuvant chemotherapy, and 67.4% receiving adjuvant chemotherapy (Table 3). This did not differ significantly by life expectancy or by chronologic age. Patterns of surgical therapy did, however, differ by life expectancy, with the most notable difference being between the rates of lumpectomy + axillary surgery (life expectancy ≥ 10 years: 70.4% vs. life expectancy < 10 years: 31.3%) vs. lumpectomy alone (life expectancy ≥ 10 years: 7.4%, life expectancy < 10 years: 31.3%) (*p* = 0.001). Adjuvant radiation rates were higher than in the HR+/HER2- group (53.5% vs. 22.1%, *p* < 0.001).

**Figure 3:**
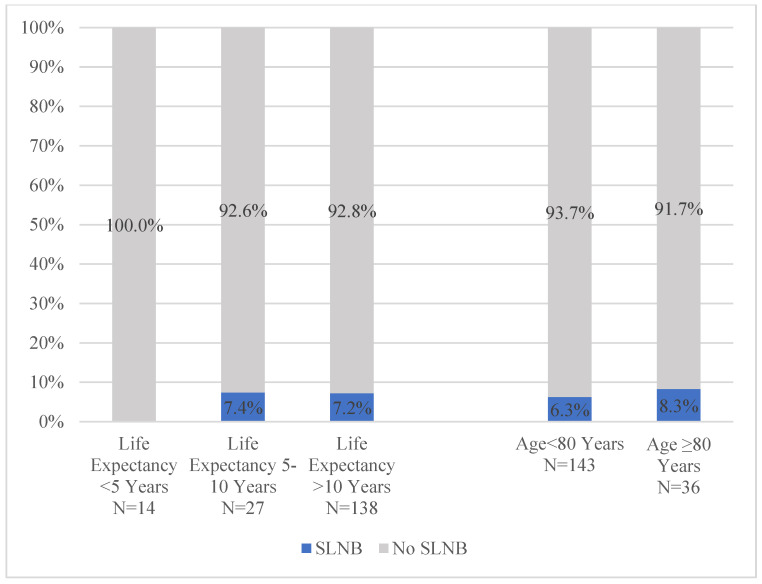
Sentinel lymph node biopsy receipt in Stage I–II lumpectomy patients ≥ 70 with HR+/HER-2− disease by life expectancy and chronologic age. *p* = 0.76 for life expectancy comparisons. *p* = 0.71 for chronologic age comparisons. Abbreviations: SLNB: sentinel lymph node biopsy.

**Figure 4:**
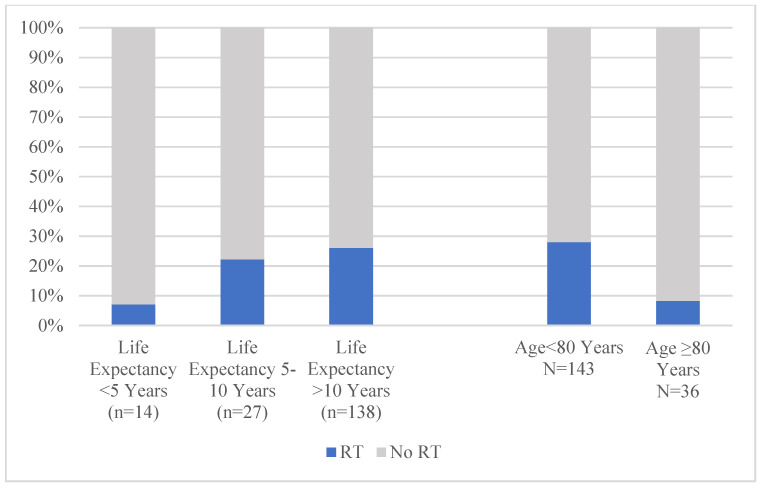
Radiation therapy receipt in Stage I–II lumpectomy patients ≥ 70 with HR+/HER-2− disease by life expectancy and chronologic age. *p* = 0.31 for life expectancy comparisons. *p* = 0.01 for chronologic age comparisons. Abbreviations: RT: radiation therapy.

In the subgroup of patients with Stage I–II HR+/HER2- disease (i.e., those in whom SLNB and RT omission should be considered), neither SLNB rates (*p* = 0.76) (Figure 3) nor RT rates (*p* = 0.31) (Figure 4) differed significantly by life expectancy.

While SLNB rates also did not differ by chronologic age (*p* = 0.71), there was a significant difference by chronologic age in RT receipt (age < 80: 28.0% vs. age > 80: 8.3%, *p* = 0.01). However, on adjusted analysis, there was no significant association between chronologic age, frailty, life expectancy, and either receipt of SLNB or RT (not shown). In more parsimonious models testing the association of life expectancy with SLNB and RT receipt, there was also no significant association between this measure of physiologic age and locoregional therapy receipt. In addition, self-reported health status was also not significantly associated with receipt of either treatment. However, omission of RT and omission of SLNB were significantly associated with each other (Table 4 and Table 5) in these models.

Adjusted analysis for the receipt of multi-modal therapy in the HER2+/TNBC subgroup similarly did not show any association between life expectancy and treatment receipt (Table 6).

## 4. Discussion

In this single-institution retrospective analysis of a prospective database, we found that rates of frailty and limited life expectancy increased with chronological age. Nonetheless, there were still frail patients with a limited life expectancy among the 70–74-year age group as well as robust patients with a life expectancy ≥ 10 years in the ≥85 year group, underscoring the heterogeneity of the aging process. While there were some differences noted by chronologic age in treatment receipt (e.g., RT receipt in both HR+/HER2- and HER-2+ and TNBC patients) in unadjusted analyses, this did not bear out in adjusted analysis. Moreover, treatment patterns largely did not differ by patient life expectancy.

Frailty, life expectancy, and chronologic age are all inter-related but, as we demonstrate here, are different constructs. Frailty, an age-related syndrome that signifies the loss of physiologic reserve and an increased vulnerability to physiologic stress [14] has been strongly associated with higher risk of treatment-related morbidity and mortality [15,16,17]. Its presence on evaluation can thus serve as a good risk assessment tool. Life expectancy, using measures that take into account multiple factors beyond chronologic age, can also be important in treatment decision making, as the oncologic benefit of certain treatments are only realized over a certain timeframe [7,8,9]. Although physiologic age should guide treatment receipt both in low-risk cancers, like stage I–II HR+/HER-2− disease, and high-risk cancers, like HER-2+ and TNBCs, in this particular cohort, there was not a clear signal that this was common practice. This is consistent with other literature demonstrating that chronologic, rather than physiologic age, can be a stronger factor in clinician decision making [18,19,20,21]. To change this practice will take the re-education of clinicians in both (1) gathering these measures of physiologic age on a regular basis, and (2) integrating these data into clinical decision making. Geriatric assessments have been recommended by large societies such as the American Society of Clinical Oncology [4] and the International Society of Geriatric Oncology [5]. Widespread implementation of geriatric assessments in oncology practice, however, has proved to be difficult; noted barriers in the literature include time costs, financial costs, and limited resources within hospital systems. However, these barriers can be addressed by leveraging non-specialists’ time (e.g., having the geriatric assessment be carried out by non-oncologist clinicians and primary care providers, and having patients self-report as many items as possible), having insurers and payers incentivize GA use, leveraging digital technologies to carry out assessments and deliver interventions, and creating oncology systems of care backed by local or national policies and institutional champions [22].

Ideally, data gathered from geriatric-specific screening tools and comprehensive geriatric assessments should be tied to changes in downstream treatment decision making. For instance, more frail individuals with limited life expectancy would be unlikely to significantly benefit from receipt of RT in early-stage HR+/HER2- disease [23], or from toxic chemotherapy regimens in the setting of higher-risk disease like TNBC [24]. However, these decisions may be difficult, as physician recommendation and patient preference may not align. Shared decision making should always be a part of cancer treatment decision making but is of higher importance in populations in which treatment guidelines are unclear and preference-sensitive decisions abound, as can often be the case in the older adult population. We report on self-reported health in this analysis, as it is one of the items captured on the Geriatric-8. Some previous literature have suggested that patients’ perception of their own health can be significantly associated with treatment patterns [25,26] and outcomes [27], but the data are mixed [28,29]. In our analysis, self-reported health status was not associated with receipt of potential overtreatment in patients with early-stage HR+/HER2- disease. However, understanding a patient’s conception of their own health status and how it may guide their preferences still needs to be understood by their treating clinicians and appropriately integrated into the final treatment decision. In addition, the lack of association between life expectancy overall and treatment receipt patterns in this analysis may demonstrate the tendency of physicians to prioritize their own subjective evaluation of a patient, which can be discordant with objective geriatric assessment [30,31].

As expected, the largest subgroup in this analysis were the HR+/HER2- patients, in whom de-escalation data exist. Trial data demonstrate that axillary surgery (consisting in modern cohorts of SLNB) and RT can be safely omitted without decrements in survival [6,7,8,9], but these practices varies significantly by institution [32]. In this particular cohort, SLNB rates are low, with less than 20% receiving any sort of axillary surgery, as are RT rates, with just over 20% receiving RT. The uptake of de-escalation practices are thus higher at our institution than reported in analysis of national datasets, in which SLNB use can be as high as 85% and RT use is 65% in patients potentially eligible for omission [11,33]. Our baseline low rates of SLNB and RT thus may have potentially obscured the associations between measures of physiologic age and receipt of these therapies.

Among patients with HER-2+ and TNBCs, chemotherapy receipt was, as expected much higher than in the HR+/HER-2 cohort. The lack of significant difference by life expectancy or chronologic age perhaps speaks to the higher risk of the disease; that is, the risk of distant disease recurrence is high enough in these subtypes to warrant treatment in all patients except for those older adults with a very short life expectancy and who are thought to be unable to bear the physiologic stress of this treatment. The low rates of chemotherapy use and adjuvant CDK 4/6 inhibitors in the HR+/HER2- group likely highlights the fact that adjuvant endocrine therapy alone is often the systemic treatment option with the best risk/benefit profile. However, while we do not have endocrine therapy adherence in this particular dataset, we do know that low rates of endocrine therapy adherence across the lifespan need to be considered when making treatment decisions [34]. While endocrine therapy was required in the trials supporting omission of SLNB and RT [7,8,9], it is possible that in early-stage HR+/HER2- patients may reasonably be able to choose between adjuvant RT and ET in the future given that the majority of the benefit of ET in this older population is with respect to lowering local recurrence risk rather than significantly lowering distant disease recurrence [35].

### Limitations

This analysis was limited by a number of factors. First, this reflects only the practice patterns and patient population of a large urban academic cancer center. Second, low numbers in certain subgroups of patients limited our ability to assess for certain associations, such as chemotherapy receipt in patients with high-risk disease, Stage II–III HER-2+ and TNBC, and introduced potential Type II error in our subgroup evaluations. Third, the low rates of SLNB and RT in our HR+/HER2- group represent our institutional practices, and could have obscured relationships between usage rates and physiologic age. Third, comorbidities are not captured in the COQD, although comorbidity evaluation is part of both the Geriatric-8 and Schonberg Index scores, and thus captured indirectly. The lack of adjustment for comorbidities, as well as domains found in a more robust geriatric assessment, such as social support and objective cognitive evaluation, limits our ability to identify associations between various aspects of frailty and contributors to physiologic age and patterns of treatment. Fourth, the COQD only captures patients who underwent surgery at our institution, and thus patients who opted for non-operative treatment are excluded from this dataset. Fifth, the overwhelming majority of this population was non-Hispanic white, and the racial and ethnic homogeneity of this population limits generalizability to more diverse populations.

## 5. Conclusions

While patients at risk of frailty and limited life expectancy are common in our population, these measures do not appear to significantly influence patient and clinician treatment decision making. Further research to establish treatment algorithms by measures of physiologic age may help to improve treatment tailoring in the older adult population.

## Figures and Tables

**Table 1:** Patient and disease characteristics by life expectancy and chronologic age.

	Total N = 272	Life Expectancy ≥ 10 Years N = 208 (76.5%)	Life Expectancy < 10 Years N = 64 (23.5%)	*p*-Value	Age <80 Years N = 215 (79.0%)	Age ≥80 Years N = 57 (21.0%)	*p*-Value
Age				<0.0001			
70–74	112 (41.2%)	104 (50.0%)	8 (12.5%)				
75–79	103 (37.9%)	75 (36.1%)	28 (43.8%)				
80–84	37 (13.6%)	25 (12.0%)	12 (18.8%)				
≥85	20 (7.4%)	4 (1.9%)	16 (25.0%)				
Frailty Status				<0.0001			0.002
Robust	168 (61.8%)	148 (71.2%)	20 (31.3%)		143 (66.5%)	25 (43.9%)	
At risk of being frail	104 (38.2%)	60 (28.8%)	44 (68.8%)		72 (33.5%)	32 (56.1%)	
Self-Reported Health				<0.0001			0.72
Better than others your age	134 (49.3%)	116 (55.8%)	18 (28.1%)		103 (47.9%)	31 (54.4%)	
As good as others your age	107 (39.3%)	84 (40.4%)	23 (35.9%)		88 (40.9%)	19 (33.3%)	
Not as good as others your age	14 (5.1%)	1 (0.5%)	13 (20.3%)		11 (5.1%)	3 (5.3%)	
Does not know	17 (6.3%)	7 (3.4%)	10 (15.6%)		13 (6.0%)	4 (7.0%)	
Race/Ethnicity				0.51			0.50
Non-Hispanic White	255 (93.8%)	197 (94.7%)	58 (90.6%)		202 (94.0%)	53 (93.0%)	
Hispanic White	1 (0.4%)	1 (0.5%)	0 (0.0%)		0 (0.0%)	1 (1.8%)	
Black (Hispanic and Non-Hispanic)	10 (3.7%)	6 (2.9%)	4 (6.3%)		8 (3.7%)	2 (3.5%)	
Asian/Pacific Islander	1 (0.4%)	1 (0.5%)	0 (0.0%)		1 (0.5%)	0 (0.0%)	
Other/Unknown	5 (1.8%)	3 (1.4%)	2 (3.1%)		4 (1.9%)	1 (1.8%)	
Tumor Grade				0.19			0.41
Grade 1	87 (32.0%)	72 (34.6%)	15 (23.4%)		70 (32.6%)	17 (29.8%)	
Grade 2	125 (46.0%)	95 (45.7%)	30 (46.9%)		101 (47.0%)	24 (42.1%)	
Grade 3	56 (20.6%)	38 (18.3%)	18 (28.1%)		40 (18.6%)	16 (28.1%)	
Unknown	4 (1.5%)	3 (1.4%)	1 (1.6%)		4 (1.9%)	0 (0.0%)	
Pathologic T Stage				0.30			0.09
T1	194 (71.3%)	153 (73.6%)	41 (64.1%)		161 (74.9%)	33 (57.9%)	
T2	33 (12.1%)	22 (10.6%)	11 (17.2%)		21 (9.8%)	12 (21.1%)	
T3	8 (2.9%)	6 (2.9%)	2 (3.1%)		6 (2.8%)	2 (3.5%)	
T4	1 (0.4%)	1 (0.5%)	0 (0.0%)		1 (0.5%)	0 (0.0%)	
Tx	1 (0.4%)	0 (0.0%)	1 (1.6%)		1 (0.5%)	0 (0.0%)	
Unknown	35 (12.9%)	26 (12.5%)	9 (14.1%)		25 (11.6%)	10 (17.5%)	
Pathologic N Stage				0.71			0.23
N0	44 (16.2%)	36 (17.3%)	8 (12.5%)		40 (18.6%)	4 (7.0%)	
N1	16 (5.9%)	14 (6.7%)	2 (3.1%)		12 (5.6%)	4 (7.0%)	
N2	3 (1.1%)	3 (1.4%)	0 (0.0%)		3 (1.4%)	0 (0.0%)	
N3	1 (0.4%)	1 (0.5%)	0 (0.0%)		1 (0.5%)	0 (0.0%)	
Nx	173 (63.6%)	128 (61.5%)	45 (70.3%)		134 (62.3%)	39 (68.4%)	
Unknown	35 (12.9%)	26 (12.5%)	9 (14.1%)		25 (11.6%)	10 (17.5%)	
Tumor Subtype				0.08			0.10
HR+/HER-2−	226 (83.1%)	179 (86.1%)	47 (73.4%)		180 (83.7%)	46 (80.7%)	
HER-2+	10 (3.7%)	6 (2.9%)	4 (6.3%)		5 (2.3%)	5 (8.8%)	
TNBC	33 (12.1%)	21 (10.1%)	12 (18.8%)		28 (13.0%)	5 (8.8%)	
Other/unknown	3 (1.1%)	2 (1.0%)	1 (1.6%)		2 (0.9%)	1 (1.8%)	
OncotypeDX Score				0.06			1.00
≤25	43 (82.7%)	40 (87.0%)	3 (50.0%)		38 (82.6%)	5 (83.3%)	
>25	9 (17.3%)	6 (13.0%)	3 (50.0%)		8 (17.4%)	1 (16.7%)	
Missing (Not performed)	220	162	58		169	51	

**Table 2:** Treatment characteristics by life expectancy in HR+/HER2- patients.

	Total N = 226	Life Expectancy ≥ 10 Years N = 179 (79.2%)	Life Expectancy < 10 Years N = 47 (20.8%)	*p*-Value	Age <80 Years N = 180 (79.7%)	Age ≥80 Years N = 46 (20.3%)	*p*-Value
Neoadjuvant Chemotherapy				1.00			1.00
Yes	2 (0.9%)	2 (1.1%)	0 (0.0%)		2 (1.1%)	0 (0.0%)	
No	224 (99.1%)	177 (98.9%)	47 (100.0%)		178 (98.9%)	46 (100.0%)	
Neoadjuvant Endocrine Therapy				0.74			0.04
Yes	14 (6.2%)	12 (6.7%)	2 (4.3%)		8 (4.4%)	6 (13.0%)	
No	212 (93.8%)	167 (93.3%)	45 (95.7%)		172 (95.6%)	40 (87.0%)	
Surgery				0.78			0.37
Lumpectomy only	171 (75.7%)	132 (73.7%)	39 (83.0%)		135 (75.0%)	36 (78.3%)	
Lumpectomy + Axillary Surgery	19 (8.4%)	17 (9.5%)	2 (4.3%)		14 (7.8%)	5 (10.9%)	
Mastectomy a	9 (4.0%)	8 (4.5%)	1 (2.1%)		6 (3.3%)	3 (6.5%)	
Mastectomy + Axillary Surgery	22 (9.7%)	18 (10.1%)	4 (8.5%)		20 (11.1%)	2 (4.3%)	
Other/unknown	5 (2.2%)	4 (2.2%)	1 (2.1%)		5 (2.8%)	0 (0.0%)	
Reconstruction ^b^				1.00			1.00
Yes	4 (12.9%)	4 (15.4%)	0 (0.0%)		4 (15.4%)	0 (0.0%)	
No	27 (87.1%)	22 (84.6%)	5 (100.0%)		22 (84.6%)	5 (100.0%)	
Adjuvant Chemotherapy				1.00			1.00
Yes	8 (3.5%)	7 (3.9%)	1 (2.1%)		7 (3.9%)	1 (2.2%)	
No	218 (96.5%)	172 (96.1%)	46 (97.9%)		173 (96.1%)	45 (97.8%)	
Adjuvant Radiation Therapy				0.24			0.02
Yes	50 (22.1%)	43 (24.0%)	7 (14.9%)		46 (25.6%)	4 (8.7%)	
No	176 (77.9%)	136 (76.0%)	40 (85.1%)		134 (74.4%)	42 (91.3%)	
Adjuvant Combination CDK 4/6 Inhibitor and Endocrine Therapy				0.59			0.59
Yes	5 (2.2%)	5 (2.8%)	0 (0.0%)		5 (2.8%)	0 (0.0%)	
No	221 (97.8%)	174 (97.2%)	47 (100.0%)		175 (97.2%)	46 (100.0%)	
Adjuvant Endocrine Therapy				0.84			0.41
Yes	182 (80.5%)	143 (79.9%)	39 (83.0%)		147 (81.7%)	35 (76.1%)	
No	44 (19.5%)	36 (20.1%)	8 (17.0%)		33 (18.3%)	11 (23.9%)	

^a^ Includes unilateral mastectomy and bilateral mastectomy; ^b^ Of patients who underwent mastectomy; Abbreviations: CDK: cyclin-dependent kinase.

**Table 3:** Treatment characteristics by life expectancy in HER2+ and TNBC patients.

	Total N = 43	Life Expectancy ≥10 Years N = 27 (62.8%)	Life Expectancy <10 Years N = 16 (37.2%)	*p*-Value	Age <80 Years N = 33 (76.7%)	Age ≥80 Years N = 10 (23.3%)	*p*-Value
Neoadjuvant Chemotherapy				1.00			1.00
Yes	19 (44.2%)	12 (44.4%)	7 (43.8%)		15 (45.5%)	4 (40.0%)	
No	24 (55.8%)	15 (55.6%)	9 (56.3%)		18 (54.5%)	6 (60.0%)	
Surgery				0.01			0.02
Lumpectomy only	7 (16.3%)	2 (7.4%)	5 (31.3%)		4 (12.1%)	3 (30.0%)	
Lumpectomy + Axillary Surgery ^a^	24 (55.8%)	19 (70.4%)	5 (31.3%)		22 (66.7%)	2 (20.0%)	
Mastectomy ^b^	5 (11.6%)	1 (3.7%)	4 (25.0%)		2 (6.1%)	3 (30.0%)	
Mastectomy + Axillary Surgery	5 (11.6%)	3 (11.1%)	2 (12.5%)		3 (9.1%)	2 (20.0%)	
Other/unknown	2 (4.7%)	2 (7.4%)	0 (0.0%)		2 (6.1%)	0 (0.0%)	
Reconstruction ^c^				0.40			1.00
Yes	1 (10.0%)	1 (25.0%)	0 (0.0%)		1 (20.0%)	0 (0.0%)	
No	9 (90.0%)	3 (75.0%)	6 (100.0%)		4 (80.0%)	5 (100.0%)	
Adjuvant Chemotherapy				0.09			0.25
Yes	29 (67.4%)	21 (77.8%)	8 (50.0%)		24 (72.7%)	5 (50.0%)	
No	14 (32.6%)	6 (22.2%)	8 (50.0%)		9 (27.3%)	5 (50.0%)	
Adjuvant Radiation Therapy				0.76			0.03
Yes	23 (53.5%)	15 (55.6%)	8 (50.0%)		21 (63.6%)	2 (20.0%)	
No	20 (46.5%)	12 (44.4%)	8 (50.0%)		12 (36.4%)	8 (80.0%)	

^a^ Includes sentinel lymph node biopsy and axillary lymph node dissection; ^b^ Includes unilateral mastectomy and bilateral mastectomy; ^c^ Of patients who underwent mastectomy.

**Table 4:** Adjusted analysis of receipt of SLNB in Stage I–II lumpectomy patients ≥ 70 with HR+/HER-2− disease.

N = 179	OR (95% CI)
Life Expectancy	
<10 Years	Ref
≥10 Years	0.81 (0.20–3.28)
Self-Reported Health	
Better than others your age	Ref
As good as others your age	0.51 (0.15–1.78)
Not as good as others your age	0.44 (0.02–10.93)
Does not know	0.29 (0.02–4.81)
Race	
White	Ref
Black	1.36 (0.13–14.02)
Asian/Pacific Islander and Others	3.19 (0.25–41.55)
Tumor Grade	
Grade 1	Ref
Grade 2	0.92 (0.24–3.50)
Grade 3	3.92 (0.72–21.26)
Pathologic T Stage	
T1	Ref
T2	1.79 (0.38–8.40)
Adjuvant Radiation Therapy	
Yes	Ref
No	0.24 (0.08–0.76)
Adjuvant Endocrine Therapy	
Yes	Ref
No	1.54 (0.40–5.94)

Events per variable: 12 events/7 covariates.

**Table 5:** Adjusted analysis of receipt of RT in Stage I–II lumpectomy patients ≥ 70 with HR+/HER-2− disease.

N = 179	OR (95% CI)
Life Expectancy	
<10 Years	Ref
≥10 Years	1.14 (0.44–2.93)
Self-Reported Health	
Better than others your age	Ref
As good as others your age	0.98 (0.47–2.06)
Not as good as others your age	0.17 (0.008–3.71)
Does not know	1.3 (0.29–5.79)
Race	
White	Ref
Black	2.21 (0.44–11.25)
Asian/Pacific Islander and Others	2.50 (0.26–23.74)
Tumor Grade	
Grade 1	Ref
Grade 2	1.33 (0.61–2.87)
Grade 3	1.00 (0.25–3.91)
Pathologic T Stage	
T1	Ref
T2	0.73 (0.23–2.37)
SLNB Receipt	
Yes	Ref
No	0.23 (0.07–0.83)
Adjuvant Endocrine Therapy	
Yes	Ref
No	0.91 (0.36–2.30)

Abbreviations: SLNB: sentinel lymph node biopsy. Events per variable: 43 events/7 covariates.

**Table 6:** Adjusted analysis of receipt of multi-modal therapy (chemotherapy/HER2+-directed therapy + surgery).

N = 43	OR (95% CI)
Life Expectancy	
<10 Years	Ref
≥10 Years	0.99 (0.21–4.61)
Pathologic T Stage	
T1	Ref
T2	0.25 (0.02–2.76)
Unknown	3.76 (0.74–18.98)

## Data Availability

Data are not available to outside researchers as this is an institutional quality dataset.

## Abbreviations

The following abbreviations are used in this manuscript:

HR	Hormone receptor
HER-2	Human epidermal growth factor receptor-2
SLNB	Sentinel lymph node biopsy
RT	Radiation therapy
TNBC	Triple negative breast cancer
COQD	Clinical Operations Quality Database
CDK	Cyclin dependent kinase
ET	Endocrine therapy
